# Evidence for Bell-Shaped Dose-Response Emetic Effects of Temsirolimus and Analogs: The Broad-Spectrum Antiemetic Efficacy of a Large Dose of Temsirolimus Against Diverse Emetogens in the Least Shrew (*Cryptotis parva*)

**DOI:** 10.3389/fphar.2022.848673

**Published:** 2022-04-04

**Authors:** Louiza Belkacemi, Yina Sun, Nissar A. Darmani

**Affiliations:** Department of Basic Medical Sciences, College of Osteopathic Medicine of the Pacific, Western University of Health Sciences, Pomona, CA, United States

**Keywords:** emesis, nucleus tractus solitarius, dorsal vagal complex, antiemetic, least shrew, temsirolimus, analogs

## Abstract

Temsirolimus is a prodrug form of sirolimus (rapamycin). With its analogs (everolimus, ridaforolimus, and rapamycin), it forms a group of anticancer agents that block the activity of one of the two mammalian targets of rapamycin (mTOR) complexes, mTORC1. We investigated the emetic potential of varying doses (0, 0.5, 1, 2.5, 5, 10, 20, and 40 mg/kg, i.p.) of temsirolimus in the least shrew. Temsirolimus caused a bell-shaped and dose-dependent increase in both the mean vomit frequency and the number of shrews vomiting with maximal efficacy at 10 mg/kg (*p* < 0.05 and *p* < 0.02, respectively). Its larger doses (20 or 40 mg/kg) had no significant emetic effect. We also evaluated the emetic potential of its analogs (5, 10, and 20 mg/kg, i.p.), all of which exhibited a similar emetic profile. Our observational studies indicated that temsirolimus can reduce the shrew motor activity at 40 mg/kg, and subsequently, we examined the motor effects of its lower doses. At 10 and 20 mg/kg, it did not affect the spontaneous locomotor activity (distance moved) but attenuated the mean rearing frequency in a U-shaped manner at 10 mg/kg (*p* < 0.05). We then determined the broad-spectrum antiemetic potential of a 20 mg/kg (i.p.) dose of temsirolimus against diverse emetogens, including selective and nonselective agonists of 1) dopaminergic D_2/3_ receptors (apomorphine and quinpirole); 2) serotonergic 5-HT_3_ receptors [5-HT (serotonin) and 2-methyl-5-HT]; 3) cholinergic M_1_ receptors (pilocarpine and McN-A-343); 4) substance P neurokinin NK_1_ receptors (GR73632); 5) the L-type calcium (Ca^2+^) channel (LTCC) (FPL64176); 6) the sarcoplasmic endoplasmic reticulum Ca^2+^ ATPase inhibitor, thapsigargin; 7) the CB_1_ receptor inverse agonist/antagonist, SR141716A; and 8) the chemotherapeutic cisplatin. Temsirolimus prevented vomiting evoked by the aforementioned emetogens with varying degrees. The mechanisms underlying the pro- and antiemetic effects of temsirolimus evaluated by immunochemistry for c-fos expression demonstrated a c-fos induction in the AP and NTS, but not DMNX with the 10 mg/kg emetic dose of temsirolimus, whereas its larger antiemetic dose (20 mg/kg) had no significant effect. Our study is the first to provide preclinical evidence demonstrating the promising antiemetic potential of high doses of temsirolimus and possibly its analogs in least shrews.

## Highlights

- Temsirolimus is a prodrug form of sirolimus (rapamycin), and its analogs evoke bell-shaped and dose-dependent increases in vomiting
- Lower doses (0.5–10 mg/kg) of temsirolimus are proemetic
- A large dose (20 mg/kg) of temsirolimus is antiemetic against diverse emetogens
- A 10 mg/kg emetic dose of temsirolimus reduced rearing and increased c-fos expression in the AP and NTS emetic zones
- A 20 mg/kg antiemetic dose of temsirolimus had no effect on either rearing or c-fos expression in the brainstem emetic loci

## 1 Introduction

Nausea and vomiting are common symptoms of various etiologies, including adverse effects of drugs (Horn 2008). These effects are the most dreaded and debilitating side effects of numerous cancer chemotherapeutics and the major cause of patient noncompliance. Emesis is a highly specific physical event that produces a rapid forceful evacuation of gastric contents in a retrograde manner from the stomach up to and out of the mouth (Quigley et al., 2001). Although many antiemetics can be effective against specific types of vomiting, frequently, they do not provide complete protection and often lack broad-spectrum antiemetic efficacy. Therefore, the search for an effective and well-tolerated prophylactic antiemetic treatment is vital for patients receiving cancer chemotherapy (Chung et al., 2011) or other therapies associated with nausea and vomiting.

The functional pathophysiology of nausea and vomiting indicates that emetic processes are controlled by a balance between the gastrointestinal enteric nervous system, the vagus, and the central nervous system (CNS) (Darmani and Ray 2009). In the case of chemotherapeutics such as cisplatin, emesis proceeds through peripheral and central pathways (Hesketh 2008; Janelsins et al., 2013). The act of vomiting may result from 1) the direct stimulation of the brainstem dorsal vagal complex (DVC) emetic nuclei including the area postrema (AP), the nucleus tractus solitarius (NTS), and the dorsal motor nucleus of the vagus (DMNX) and/or 2) the indirect stimulation of the DVC via peripheral activation of emetic loci such as the neurons of the enteric nervous system (ENS) and release of emetic neurotransmitters from the gastrointestinal enterochromaffin (EC) cells which subsequently activate the gastrointestinal vagal afferents to the brainstem (Darmani and Ray 2009), (Ray et al., 2009a) (Babic and Browning 2014). In addition, emetogens can gain access to the brainstem through blood-borne mechanisms as the NTS lacks an effective blood–brain barrier. The emetic response involves multiple neurotransmitters such as dopamine, serotonin, and substance P, all of which have been recognized as mediators of chemotherapy-evoked vomiting (Hesketh 2008; Singh et al., 2016). The emetic receptors for these neurotransmitters, namely, dopamine D_2_, serotonin 5-HT_3_, and neurokinin NK_1_, are implicated in the pathophysiological mechanisms of chemotherapy-induced vomiting.

**FIGURE 1:**
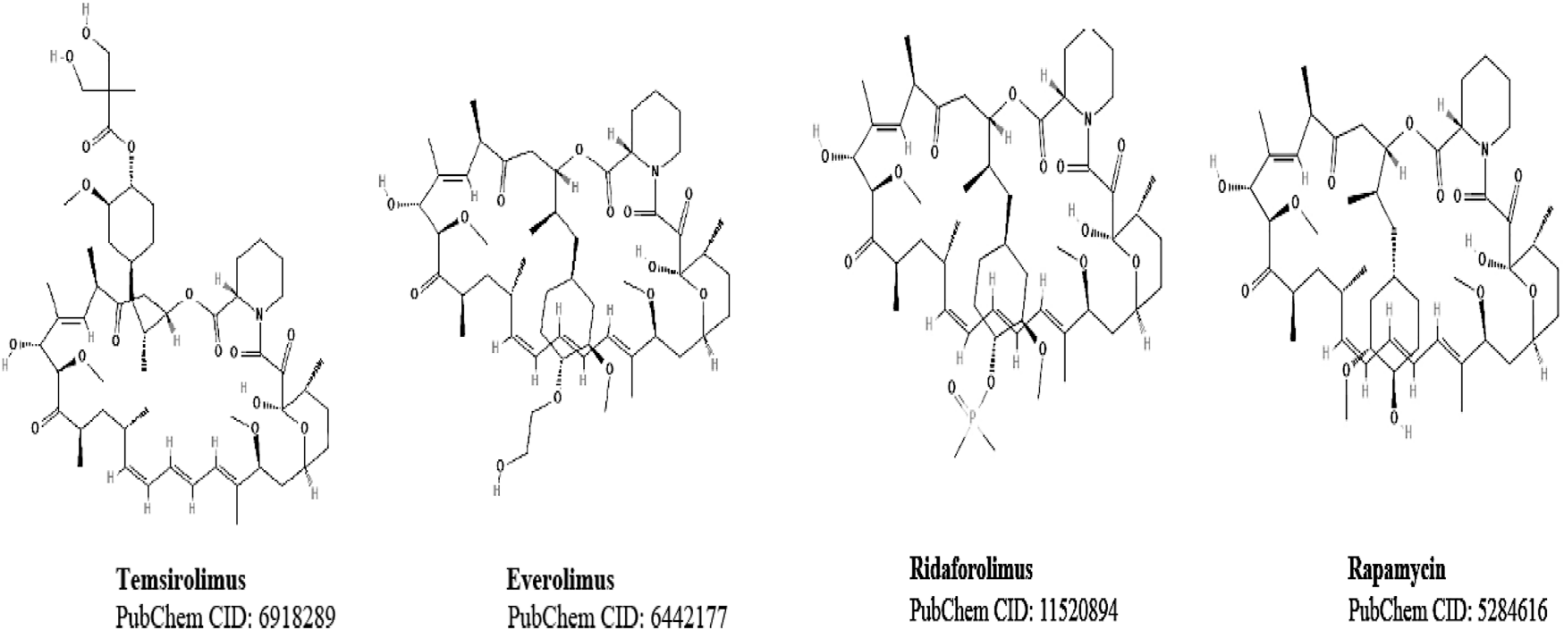
Structure of temsirolimus and its analogs.

In recent years, the mammalian target of rapamycin (mTOR) inhibitors, rapamycin, and its analogs (Figure 1) have become promising therapeutic drugs against cancer because of mTOR’s role in tumor progression. mTOR kinase acts on two functionally distinct complexes: mTOR complex 1 (mTORC1) and 2 (mTORC2) (Meng and Zheng 2015; Kim et al., 2017). The clinical importance of rapamycin and its analogs is because of these agents’ ability to block mTORC1 activity. Among the rapamycin analogs, temsirolimus, everolimus, and ridaforolimus have been clinically applied in mono- and combination therapies against different types of cancers. More recently, second-generation mTOR inhibitors (Meng and Zheng 2015), including combined inhibitors of mTORC1 and mTORC2 (Garcia-Echeverria 2010; Zhang and Zheng 2012) and dual phosphatidylinositol-3-kinase (PI3K)/mTOR suppressors, have been described (Tarantelli et al., 2020). Detrimental gastrointestinal side effects such as nausea and vomiting (Lebranchu et al., 2009) have been reported in patients taking such drugs. Indeed, nausea and vomiting have been reported, respectively, in about 30–40% of patients treated with temsirolimus (Malizzia and Hsu 2008; Hadoux et al., 2010) and 49–68% in patients treated with BGT226, a potent and dual phosphoinositide 3-kinase (PI3K)/mTOR inhibitor (Markman et al., 2012). Despite their clinical benefits, the role of mTOR blockers in the incidence of nausea and vomiting remains largely underexplored. Similarly, rapamycin and its analogs’ effects on motor behaviors also remain unknown.

Considering the present knowledge gap, we initially studied the potential emetic dose-response effects of the administration of varying doses of temsirolimus (0.5–40 mg/kg, i.p.) in the least shrew model of emesis. Temsirolimus exhibited a bell-shaped dose-response effect, with lower doses (0.5–10 mg/kg) being proemetic and larger doses such as 20–40 mg/kg having little to no emetic activity. These observations also indicated that the 40 mg/kg dose of temsirolimus may reduce locomotor activity. Locomotor tests revealed that although 10 and 20 mg/kg (i.p.) doses of temsirolimus had no significant effect on the total distance moved by shrews, the frequency of rearing behavior was significantly attenuated at its 10 but not 20 mg/kg dose. To determine whether rapamycin’s analogs (Figure 1; everolimus, ridaforolimus, and rapamycin) may have a similar emetic profile, we investigated the emetic effects of their low (5 and 10 mg/kg) and high (20 mg/kg) doses on the incidence of vomiting. In addition, we investigated the antiemetic potential of a 20 mg/kg (i.p.) dose of temsirolimus against the well-investigated fully effective emetic doses of the following diverse emetogens: 1) the dopamine D_2/3_ receptor nonselective agonist apomorphine and the more selective D_2_ receptor agonist quinpirole (Darmani et al., 1999; Darmani and Crim 2005); 2) the nonselective serotonin 5-HT_3_ receptor agonist 5-HT and its more selective agonist 2-methyl-5-HT (Darmani et al., 2014); 3) the cholinergic M_1_ receptor nonselective agonist pilocarpine, and its more selective agonist McN-A-343 (Beleslin et al., 1989; Darmani et al., 2014); 4) the selective substance P neurokinin NK_1_ receptor agonist GR73632 (Darmani et al., 2008; Zhong et al., 2019); 5) the selective L-type calcium (Ca^2+^) channel agonist FPL64176 (Zhong et al., 2018); 6) the sarcoplasmic/endoplasmic reticulum (ER) Ca^2+^ ATPase inhibitor thapsigargin (Zhong et al., 2016); 7) the selective cannabinoid CB_1_ receptor inverse agonist/antagonist SR141716A (Darmani et al., 2003); and 8) the chemotherapeutic agent cisplatin (Darmani et al., 2009). Our findings indicated that the 20 mg/kg dose of temsirolimus markedly suppressed vomiting to varying degrees caused by all tested emetogens. The mechanisms underlying the pro- and antiemetic effects of temsirolimus were evaluated by immunochemistry for c-fos expression demonstrated a c-fos induction in the AP and NTS, but not DMNX with a 10 mg/kg (i.p.) proemetic dose of temsirolimus, whereas its larger antiemetic dose (20 mg/kg, i.p.) had no effect on baseline levels of c-fos expression in all three zones. Overall, our study provided evidence for proemetic effects of low doses of temsirolimus and its antiemetic potential at larger (e.g., 20 mg/kg) doses.

## 2 Materials and Methods

### 2.1 Animals

Adult least shrews between 45 and 60 days old and weighing between 4 and 6 g from the Western University of Health Sciences Animal Facilities were used. The shrews were housed in groups of 5–10 on a 14:10 light/dark cycle and fed an ad libitum diet. Our animal handling protocols were based upon a prior emesis investigation (Darman et al., 1999; Zhong et al., 2016). Animal experiments were conducted in accordance with the principles and procedures of the National Institutes of Health Guide for the Care and Use of Laboratory Animals. All protocols were approved by the Institutional Animal Care and Use Committee of Western University of Health Sciences (protocol number R20IACUC018). All efforts were made to minimalize the animals’ pain and to diminish the number of animals used in the experiments. All experiments were carried out between 8:00 a.m. and 17:00 p.m.

### 2.2 Chemicals

Temsirolimus, everolimus, apomorphine HCl, GR73632, SR141716A, thapsigargin, and FPL64176 were purchased from Tocris (Minneapolis, MN). Ridaforolimus and rapamycin were acquired from MedChemExpress and Calbiochem, respectively. Quinpirole HCl, serotonin HCl (5-HT), 2-methyl-serotonin maleate salt (2-methyl-5-HT), pilocarpine HCL, McN-A-343, and cisplatin (cis-platinum (II) diamine dichloride (Pt (NH_3_)_2_)Cl_2_) were obtained from Sigma/RBI. Apomorphine, quinpirole, serotonin, 2-methy-5-HT, McN-A-343, GR73632, pilocarpine, and cisplatin were dissolved in distilled water. Thapsigargin was dissolved in 10% DMSO (Sigma) in water. mTOR inhibitors and FPL64176 were dissolved in DMSO and then diluted with three volumes of distilled water to a final DMSO concentration of 25%. SR141716A was dissolved in a 1:1:18 solution of ethanol, emulphor (EL-620, a polyoxyethylated vegetable oil, GAF Corporation, Linden, NJ), and 0.9% saline. All drugs were administered at a volume of 0.1 ml/10 g of body weight. The doses and routes used for the emetogens were based upon previous publications from our laboratory (Darmani et al., 2019; Darmani et al., 2020; Zhong and Darmani 2020).

### 2.3 Experimental Protocols

#### 2.3.1 Emesis Studies

On the day of the experiment, the shrews were transported to the experimental room from the animal facility, weighed, transferred to 20 × 18 × 21 cm clean clear plastic individual cages, and permitted to acclimate for 1 h during which daily food was withdrawn. Drug-naïve male and female shrews were randomly assigned to the control and the experimental groups irrespective of their cage of origin. The shrews were given four meal worms (*Tenebrio* sp.) every 30 min before the administration of emetogens, to aid in identifying wet vomits as described previously (Darmani et al., 1999).

To determine the dose-response emetic effect of temsirolimus, different groups of least shrews were pretreated intraperitoneally (i.p.) at 0 min with either the vehicle or varying doses of temsirolimus (0.5, 1, 2.5, 5, 10, 20, or 40 mg/kg, respectively; *N* = 9–14 shrews within a group). The number of evoked vomits were counted for the next 30 min. In additional experiments, different groups of shrews were pretreated at 0 min with an i.p. injection of either vehicle (25% DMSO in sterile deionized distilled water; *N* = 8–10 shrews within a group) or varying doses (5, 10, or 20 mg/kg i.p.) of different analogs of temsirolimus as follows: everolimus (*N* = 7–9 shrews within a group), ridaforolimus (*N* = 7, 8 shrews within a group), or rapamycin (*N* = 6–10 shrews within a group). The number of evoked vomits were counted for the next 30 min as described previously. Each shrew was used once and euthanized using 3% isoflurane at the end of the experiment.

#### 2.3.2 Locomotor Activity and Rearing Behavior

Emetic dose-response studies indicated that temsirolimus may attenuate spontaneous locomotor activity at 40 mg/kg dose, and therefore we evaluated its potential motor-suppressive effects (i.e., total distance moved and rearing behavior) at 10 and 20 mg/kg (i.p.) doses. The total distance moved reflects the ability of a test drug to interfere with the normal motor functioning after administration, whereas rearing correlated to environmental novelty and is consistent with exploratory information-gathering (Lever et al., 2006). The locomotion analysis and behavior recognition system, Ethovision (version XT 9) by Noldus Information Technology (Costerweg, Netherlands) was used. The parameters of Ethovision were set to record two motor activities: 1) total distance moved in meters (spontaneous locomotor activity) and 2) rearing frequency. Rearing was recorded when a 5% reduction in the shrew body surface area occurred when a shrew stood upright as seen by the overhead video camera. On the day of the test, the shrews were brought in their home cages to the experimental room and were allowed, for 1 h, to acclimate to a semi-dark environment, which was required for the computerized Ethovision System to work efficiently. Each shrew was further acclimated in an empty white plastic dummy observation cage (27.5 × 27.5 × 28 cm) for 1 h before testing. Different groups of shrews were injected with either vehicle (25% DMSO, i.p., *N* = 9 within a group) or varying doses of temsirolimus (10 or 20 mg/kg, i.p., *N* = 8 within a group). Each shrew was individually placed in a white observation cage of the same dimension, and the two motor parameters were recorded for 30 min by an overhead camera and data were analyzed using the Noldus software. When changing to another shrew, the chamber was thoroughly cleaned with 70% ethanol and dried to eliminate animal odors between test sessions. Each shrew was used once and euthanized using isoflurane at the end of the experiment.

#### 2.3.3 Antiemetic Effects of a High Dose of Temsirolimus (20 mg/kg) Against Fully Effective Emetic Doses of Well-Investigated Diverse Emetogens

A 20 mg/kg dose of temsirolimus was tested against an array of emetogens. Thus, different groups of shrews were pretreated at 0 min with an injection of either vehicle (25% DMSO, i.p.) or 20 mg/kg temsirolimus. At 30 min, different groups of pretreated shrews received a fully effective emetic dose of one of following emetogens: 1) a nonselective dopamine D_2/3_ receptor agonist, apomorphine (2 mg/kg, i.p.; *N* = 6, 7 within a group), or the more selective D_2_ receptor agonist quinpirole (2 mg/kg, i.p.) (*N* = 8 within a group) (Darmani et al., 1999; Darmani and Crim 2005); 2) a peripherally acting nonselective 5-HT_3_ receptor agonist, 5-HT (5 mg/kg, i.p.; *N* = 6 within a group); or a centrally/peripherally acting and more selective 5-HT_3_ receptor agonist, 2-methyl-5-HT (5 mg/kg, i.p.; *N* = 6 within a group) (Darmani et al., 2014); 3) a nonselective muscarinic agonist, pilocarpine (2 mg/kg, i.p.; *N* = 5 within a group) (Zhong et al., 2014), or a more selective muscarinic M_1_ receptor agonist, McN-A-343 (2 mg/kg, i.p., *N* = 10 within a group); 4) an NK_1_ receptor-selective agonist, GR73632 (5 mg/kg, i.p.; *N* = 7–9 within a group) (Darmani et al., 2008; Zhong et al., 2019); 5) an L-type Ca^2+^ channel (LTCC) agonist, FPL64176 (10 mg/kg, i.p.; *N* = 5,6 shrews within a group) (Zhong et al., 2018); 6) a sarco/endoplasmic reticulum (ER) Ca^2+^ ATPase inhibitor (SERCA), thapsigargin (0.5 mg/kg, i.p.; *N* = 5, 6 within a group) (Zhong et al., 2016), 7) a cannabinoid CB_1_ receptor antagonist/inverse agonist SR141716A (20 mg/kg, i.p.) (*N* = 7–11 within a group); or 8) cisplatin (10 mg/kg, i.p.; *N* = 7, 8 within a group) (Darmani et al., 2013)^,^ (Darmani et al., 2015). Each shrew was watched for 30 min (or in the case of cisplatin, for 2 h) to record the frequency of vomits, to determine the mean frequency of vomits and percentage of animals vomiting. Each shrew was used once and euthanized using isoflurane at the end of the experiment.

#### 2.3.4 c-fos Immunofluorescence Staining

Different groups of shrews (*N* = 4 within a group) were treated with either vehicle (25% DMSO, i.p.) or temsirolimus (10 or 20 mg/kg i.p.). After 30 min, the shrews were anesthetized with isoflurane and perfused with ice cold 4% paraformaldehyde in pH 7.4, 0.1 M phosphate-buffered saline (PBS) for ten min. The brains were removed and cryoprotected with 30% sucrose in 0.1 M PBS overnight and sectioned on a freezing microtome (Leica, Bannockburn) into 20-μm sections. The sections were observed with a light microscope and those harboring the whole DVC were subjected to immunostaining. The sections were blocked with 0.1 M PBS containing 10% donkey serum and 0.3% Triton X-100 after which they were incubated overnight at 4°C with rabbit anti-c-fos polyclonal antibody (1:4,000, Abcam, San Diego) in the blocking buffer. The next day, the sections were washed three times (ten min each) in PBS and incubated in Alexa Fluor 594 donkey anti-rabbit IgG (1:500, Invitrogen, Eugene) secondary antibodies. The nuclei of the cells were counterstained with DAPI (Vector Laboratories, Burlingame). Images were acquired under a Zeiss confocal microscope with Zen software using ×20 objective. c-fos-immunofluorescent cell nuclei in each region (AP, NTS, or DMNX) were counted using ImageJ as described previously (Ray et al., 2009b; Zhong et al., 2018). For each region, two sections at close intervals were counted for each animal. The means of each region per animal were further compared.

### 2.4 Statistical Analysis

Assuming that type 1 error rate was set at 0.05, sample size estimates for behavioral studies were based on a power of 80% to detect a 30% change between the control and treated groups (assuming an expected standard deviation of 20% of mean values). This analysis resulted in a requirement for eight animals in each group. Statistical analysis was performed using GraphPad Prism 6.04. The vomiting frequency data were analyzed using the Kruskal–Wallis (KW) non-parametric one-way analysis of variance (ANOVA) followed by Dunnett’s post hoc test and expressed as the mean ± SEM. The percentage of animals vomiting across groups at different doses was compared using the Chi-square test. The total distance moved and rearing were compared with an ordinary ANOVA test followed by Bonferroni’s post hoc test. The comparison of latency values obtained with temsirolimus and rapamycin injected at 5, 10 and 20 mg/kg doses were presented as the fold change of mean latency of temsirolimus (sec) over mean latency of rapamycin (sec).The vomiting frequency between the two groups was analyzed using an unpaired *t*-test and Chi-square test for the percentage of animals vomiting. The c-fos-positive cells among the groups were compared by an ordinary ANOVA followed by Dunn’s post hoc test. *p* < 0.5 was considered statistically significant.

## 3 Results

### 3.1 Dose-Response Emetic Effects of Temsirolimus and Its Analogs

**FIGURE 2:**
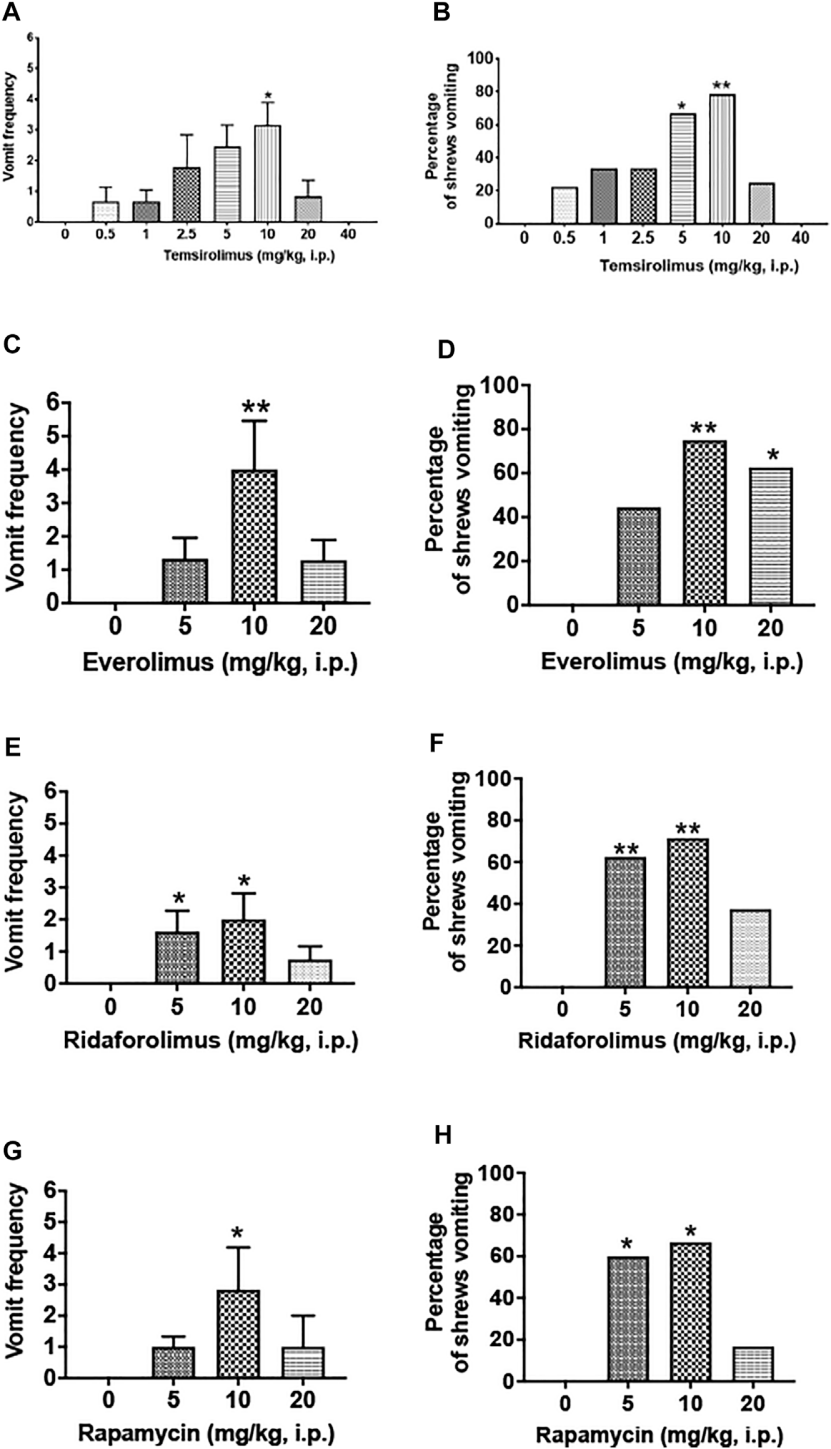
Emetic effects of varying doses of temsirolimus and its analogs in the least shrew. Different groups of shrews received either an injection of corresponding vehicle (25% DMSO, i.p.) or varying cited doses of temsirolimus or its analogs everolimus, ridaforolimus, and rapamycin. Emetic parameters were recorded for 30 min post-emetic injection. Graphs **(A,B)** respectively represent the mean frequency of vomiting (±SEM) and the percentage of shrews vomiting following temsirolimus administration. Similarly, graphs in **(C,D)**, **(E,F),** and **(G,H),** respectively, show corresponding emetic parameters for everolimus, ridaforolimus, and rapamycin. For each of the aforementioned experiment, significance in the frequency of vomiting was obtained using a Kruskal–Wallis non-parametric one-way ANOVA followed by Dunnett’s post hoc test and expressed as the mean ± SEM. Chi-square test was used to determine significant differences in the percentage of shrews vomiting relative to the corresponding vehicle control groups (25% DMSO). Significant differences are indicated as *p < 0.05; **p < 0.01.

We initially assessed the emetic potential of temsirolimus in the least shrew (Figure 2). Temsirolimus (0, 0.5, 1, 2.5, 5, 10, 20, or 40 mg/kg, i.p.) increased both the frequency of emesis and the percentage of shrews vomiting in a bell-shaped and dose-dependent manner (Figure 2 A and B). A Kruskal–Wallis (KW) non-parametric ANOVA test showed that relative to the vehicle-pretreated control group, temsirolimus significantly increased the mean vomiting frequency [(KW (7, 70) = 24.87; *p* < 0.001)] with a maximum (3.14 ± 0.74) at 10 mg/kg (*p* < 0.01) (Figure 1A). At its 20 mg/kg dose, few vomits were observed, whereas at 40 mg/kg temsirolimus no emesis occurred (Figure 2A). The chi-square test indicated that the percentage of animals exhibiting emesis in response to temsirolimus also increased in a bell-shaped and dose-dependent fashion [(χ^2^ (7, 70) = 25.34, *p* = 0.007)]. Significant increases in the percentage of shrews vomiting were observed at 5 and 10 mg/kg doses (66.7 and 78.6%; *p* = 0.015 and 0.002, respectively) (Figure 2B). However, only 25% of the shrews vomited at the 20 mg/kg dose and none vomited at 40 mg/kg (Figure 2B), indicating higher doses of temsirolimus may behave as an antiemetic.

We then extended our investigation to the emetic potential of temsirolimus analogs, everolimus, ridaforolimus, and rapamycin. A similar picture emerged in that each paralog at 10 mg/kg dose caused significant increases in both the vomiting frequency and number of shrews vomiting, whereas their larger doses generally evoked less vomiting and in fewer animals (Figure 2 C—H).

### 3.2 Open-Field Locomotor Studies

**FIGURE 3:**
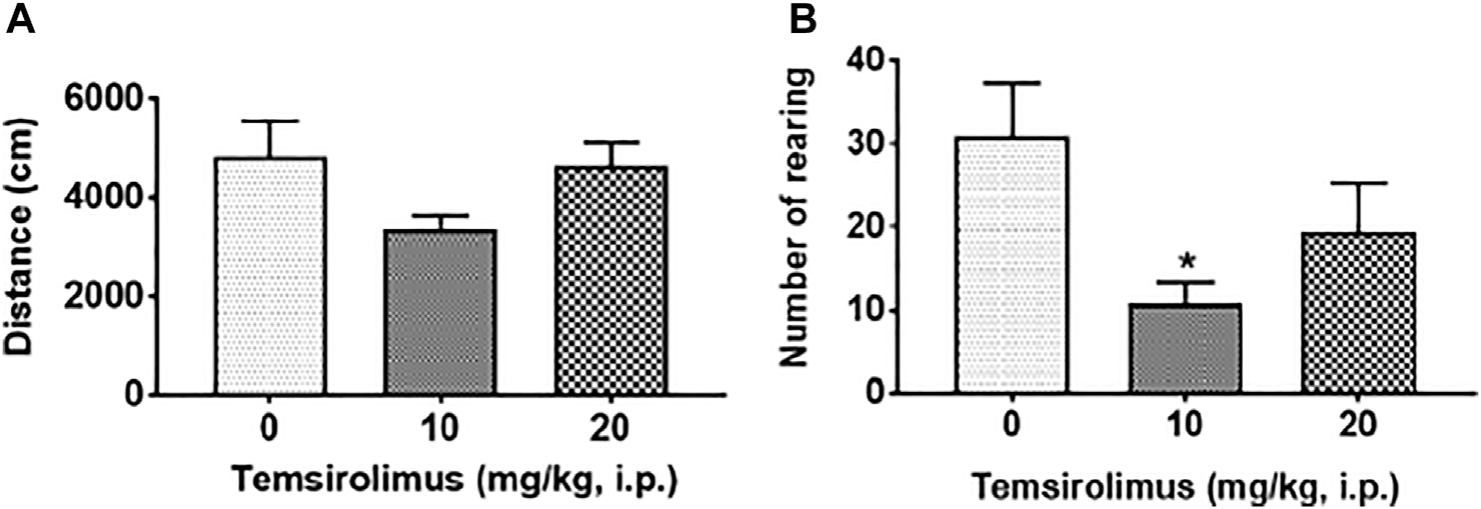
Effect of temsirolimus on open-field locomotor behaviors. The dose-response effects of temsirolimus on **(A)** locomotor activity (distance moved) and **(B)** rearing frequency in different groups of least shrews treated with either vehicle (25% DMSO, i.p.) or temsirolimus (10 or 20 mg/kg, i. p). At 0 time, shrews received vehicle or temsirolimus and the aforementioned motor behaviors were recorded for 30 min post injection by a computerized video tracking, motion analysis, and behavior recognition system (Ethovision). Significant difference relative to the corresponding 25% DMSO, i.p. vehicle group is indicated as *p < 0.05.

Figures 3 A, B show the effects of vehicle and temsirolimus (10 and 20 mg/kg, i.p.) on the locomotor activities of shrews in an open-field environment. A one-way ANOVA analysis demonstrated that both tested doses of temsirolimus had no significant effect on the total distance moved by the shrews in the 30 min observation period (*p* = 0.14; Figure 3A). In contrary, the frequency of rearing behavior was significantly attenuated in a U-shaped manner by its 10 mg/kg dose. Indeed, while in the vehicle-treated control shrews the average value for the frequency of rearing was 30.89 ± 6.47 (Figure 3B), the mean frequency of rearing decreased to 10.75 ± 2.63 (65.20%; *p* = 0.03 versus vehicle) (Figure 3B) with 10 mg/kg temsirolimus. At the 20 mg/kg dose of temsirolimus, the frequency of rearing was no longer significant with a value of 19.50 ± 5.73 (36.87%; *p* = 0.28 versus vehicle) (Figure 3B).

In the subsequent drug interaction studies, we chose the 20 mg/kg dose of temsirolimus for the evaluation of its antiemetic potential as it did not significantly affect open-field locomotor parameters.

In addition, we compared the latency values obtained with either temsirolimus or rapamycin injected at 5, 10, and 20 mg/kg doses. Latency is the period of time taken to record the first vomit for each shrew following the injection of an emetogen. An intraperitoneal injection of temsirolimus or rapamycin at a 5 mg/kg dose had greater latency than that obtained after i.p. administration of either drug at a 10 mg/kg (i.p.) dose (Supplementary Table S2). In addition, the latency value of temsirolimus was 0.6 times less than that of rapamycin at the 5 mg/kg dose. Intraperitoneal administration of 10 mg/kg temsirolimus or rapamycin produced similar latency values. The i.p. injection of 20 mg/kg temsirolimus increased the vomit latency by 4.5 times versus rapamycin (Supplementary Table S2).

### 3.3 Temsirolimus (20 mg/kg) Suppresses Vomiting Evoked by Diverse Emetogens

**FIGURE 4:**
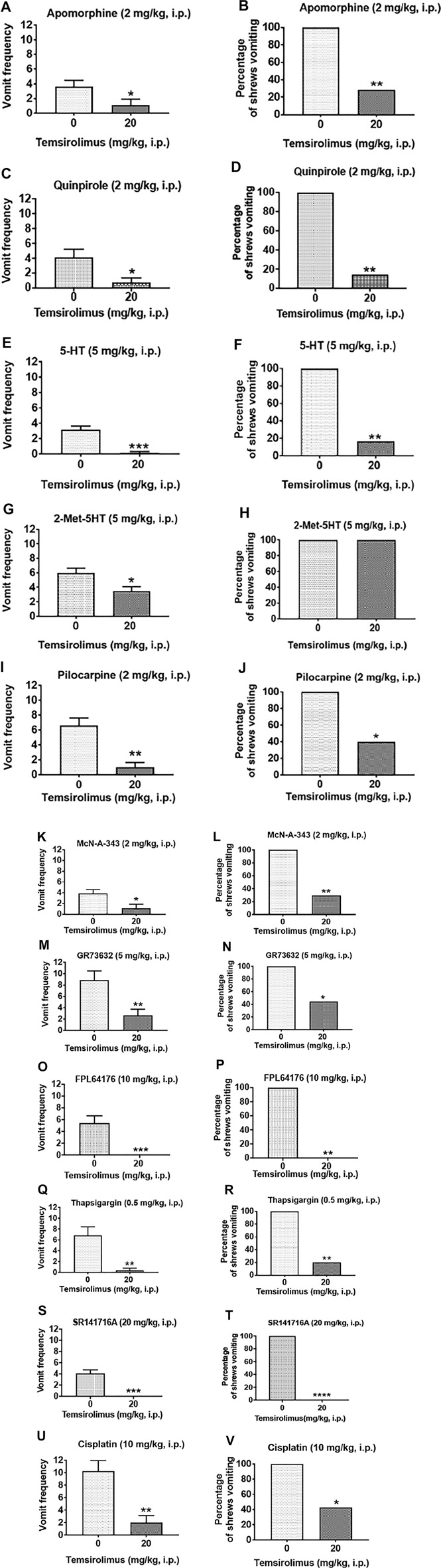
Broad-spectrum antiemetic potential of temsirolimus. Different groups of shrews were pretreated with either temsirolimus (20 mg/kg, i.p.) or its vehicle (i.p.) Thirty min prior to administration of a maximally effective dose of diverse emetogens known to evoke vomiting in all tested shrews. To varying degrees temsirolimus reduced vomiting in response to the cited emetogens. Data are presented as the mean frequency of vomits (±SEM) (graphs **(A,C,E,G,I,K,M,O,Q,S,U)**) and the percentage of shrews vomiting (graphs **(B,D,F,H,J,L,N,P,R,T,V)**) for the following emetogens respectively: apomorphine, quinpirole, 5-HT, 2-Me-5-HT, pilocarpine, McN-A-343, GR73632, FPL64176, thapsigargin, SR141716A, and cisplatin. Significant differences relative to the temsirolimus vehicle control group are indicated as *p < 0.05; **p < 0.005; ***p < 0.001; ****p < 0.0001.

The results are described and summarized in Supplementary Table S1. Temsirolimus at 20 mg/kg (i.p.) significantly suppressed apomorphine (2 mg/kg, i.p.)-induced vomiting as indicated by the emetic parameters, vomit frequency (68.85% reduction; *p* = 0.04) (Figure 4A; Supplementary Table S1), and the percentage of shrews vomiting (71.43% reduction; *p* = 0.008) (Figure 4B; Supplementary Table S1). Similarly, temsirolimus (20 mg/kg, i.p.) significantly decreased both the vomiting frequency induced by quinpirole (81.80% reduction; *p* = 0.01) (Figure 4C; Supplementary Table S1) and the percentage of shrews vomiting (75.0% reduction; *p* = 0.002) (Figure 4D; Supplementary Table S1). Also, temsirolimus at 20 mg/kg (i.p.) significantly suppressed the frequency of vomiting (94.73% reduction; *p* = 0.0001 and 41.2% reduction; *p* = 0.01, respectively) induced either by 5-HT (5 mg/kg, i.p.) or the more selective (2-methyl-5-HT) agonist of 5-HT_3_ receptors (Figure 4E,G; Supplementary Table S1). Temsirolimus also reduced the percentage of animals vomiting (83.33% reduction; *p* = 0.003) in response to 5-HT (5 mg/kg, i.p.) but not after the 2-methyl-5-HT challenge (0% reduction; *p* > 0.05) (Figure 4F,H; Supplementary Table S1). Moreover, the same dose of temsirolimus attenuated emesis evoked either by the nonselective cholinergic M_1_R agonist pilocarpine (2 mg/kg, i.p.), or the more selective M_1_R cholinergic agonist McN-A-343 (2 mg/kg, i.p.). In fact, the vomiting frequency (84.48%; *p* < 0.002 and 71.80%; *p* = 0.01, respectively) (Figure 4I,K; Supplementary Table S1) and the percentage of shrews vomiting (60%; *p* = 0.04 and 70%; *p* = 0.001, respectively) (Figure 4J,L; Supplementary Table S1) were significantly reduced.

Next, temsirolimus (20 mg/kg, i.p.) was tested against the selective substance P neurokinin NK_1_ receptor agonist GR73632 (5 mg/kg, i.p), which significantly reduced both the evoked vomiting frequency (69.96% reduction; *p* = 0.006) (Figure 4M; Supplementary Table S1) and the percentage of shrews vomiting (55.56%; *p* < 0.05) (Figure 4N; Supplementary Table S1). Similarly, temsirolimus (20 mg/kg, i. p) significantly attenuated FPL64176 (10 mg/kg, i.p.)-stimulated vomiting as indicated by the emetic parameters, vomiting frequency (100% reduction; *p* = 0.001) (Figure 4O O; Table S1), and the percentage of shrews vomiting (100%; *p* = 0.003) (Figure 4P; Supplementary Table S1). Temsirolimus (20 mg/kg, i.p.) also attenuated thapsigargin-evoked vomiting as indicated by significant reductions in the vomiting frequency (94.14% reduction; *p* < 0.01) (Figure 4Q; Supplementary Table S1) and the percentage of shrews vomiting (80.0%; *p* = 0.006) (Figure 4R; Supplementary Table S1). When temsirolimus (20 mg/kg, i.p.) was assessed against an emetic dose of SR141716A (20 mg/kg, i.p.), it fully protected the shrews from vomiting as revealed by both the evoked vomit frequency (100% reduction; *p* = 0.0001) (Figure 4S; Table S1) and the percentage of shrews vomiting (100%; *p* < 0.0001) (Figure 4T; Supplementary Table S1). Finally, temsirolimus (20 mg/kg, i.p.) significantly reduced the acute phase of cisplatin-induced vomiting as indicated by the emetic parameters, vomiting frequency (*p* = 0.002) (80.48% reduction; *p* = 0.002) (Figure 4U; Table S1), and the percentage of shrews vomiting (57.14% reduction; *p* = 0.02) (Figure 4V; Supplementary Table S1).

### 3.4 Differential c-fos Immunoreactivity After Injection of Varying Doses of Temsirolimus

**FIGURE 5:**
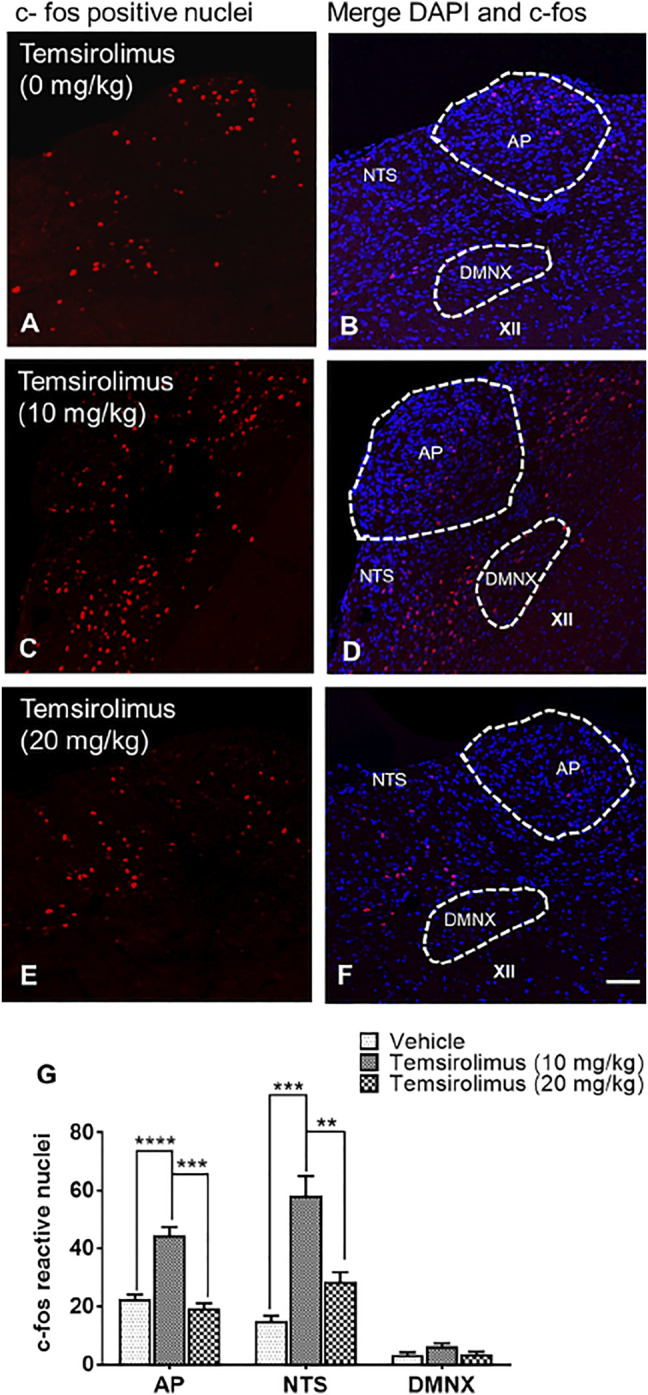
Immunohistochemical analysis of c-fos induction in the shrew brainstem emetic nuclei following administration of either 0, 10, or 20 mg/kg temsirolimus. Brainstem sections (20 μm) were prepared from different groups of shrews, including **(A)** vehicle-treated control and **(C)** 10 mg/kg (i.p.) and **(E)** 20 mg/kg (i.p.) dose of temsirolimus. Sections were immunolabeled with c-fos antibody overnight followed by Alexa red conjugated donkey anti-rabbit secondary antibody incubation. **(A,C,E)** Representative 20x images of brainstem slice showing c-fos immunoreactivity in the area postrema (AP), the nucleus of the solitary tract (NTS), and the dorsal motor nucleus of the vagus (DMNX). **(B,D,F)** Representative sections with nuclei stained with DAPI. Scale bar, 50 μm. **(G)** c-fos-immunoreactive nuclei counts shown as mean ± SEM of *N* = 4 shrew brainstems per group. **p < 0.01; ***p < 0.001, ****p < 0.0001 versus vehicle control group (25% DMSO), one-way ANOVA followed by Dunn’s post hoc test.

To determine whether temsirolimus caused c-fos induction relative to the vehicle-treated control group, we tested temsirolimus at a maximally effective emetic dose of 10 and a less effective dose of 20 mg/kg (i.p.). In line with the behavioral results, the 10 mg/kg (Figure 5 C, D, G) but not 20 mg/kg dose of temsirolimus (Figure 5 E, F, G), caused to a marked induction in c-fos in the brainstem DVC emetic nuclei (AP and NTS), but not in DMNX (Figure 5 C—F, G), compared with the non-vomiting vehicle controls (Figure 5 A, B). Indeed, in the vehicle-treated control shrews, the average values for the c-fos-positive cell nuclei were 22.6 ± 1.6, 15.0 ± 1.9, and 3.3 ± 0.9 in the AP, NTS, and DMNX, respectively (Figure 5G). Following the vomiting induced by 10 mg/kg temsirolimus, the mean numbers of the c-fos-immunoreactive cell nuclei were increased to 44.7 ± 2.7 in the AP (*p* = 0.0001 versus vehicle), 58.3 ± 6.9 in NTS (*p* = 0.0002 vehicle), and 6.2 ± 1.2 in DMNX (*p* = 0.2 versus vehicle) (Figure 5G). When 20 mg/kg temsirolimus was injected, the mean numbers of the c-fos-immunoreactive cell nuclei did not increase significantly, 19.3 ± 1.9 in the AP (*p* = 0.53 versus vehicle), 28.5 ± 3.3 in NTS (*p* = 0.14 versus vehicle), and 3.3 ± 0.9 in DMNX (*p* = 0.99 versus vehicle) (Figure 5G).

## 4 Discussion

### 4.1 Significance of the Present Study

Over the last decade, many discoveries have shown that the mTOR pathway is activated in a wide variety of cellular processes and is deregulated in human diseases such as cancer (Laplante and Sabatini 2009). These observations led to broad scientific and clinical interests in mTOR as reflected by the growing use of mTOR inhibitors (rapamycin and its analogs) in pathological settings, including the treatment of solid tumors. While these inhibitors are widely known to induce emesis in patients, so far, no basic evidence is available in vomit-competent animal models of vomiting to support their reported emetic capacity in the clinical setting. In the present study, we investigated the emetic/antiemetic potential of temsirolimus in the least shrew animal model of emesis. Temsirolimus and its analogs increased emetic parameters in a bell-shaped and a dose-dependent manner. In fact, the maximally effective emetic dose of temsirolimus (10 mg/kg, i.p.) not only caused significant increases in the mean vomiting frequency and percentage of animals vomiting, but also significantly reduced rearing behavior. Vomiting parameters and rearing behavior returned to baseline values with the 20 mg/kg temsirolimus dose. In addition, we found a significantly higher c-fos expression in the NTS and DMNX brainstem emetic loci of least shrews treated with the 10 mg/kg temsirolimus dose, but not with its 20 mg/kg dose. Importantly, the 20 mg/kg dose of temsirolimus blocked vomiting evoked by diverse emetogens acting centrally, peripherally, or both. Collectively, we have provided direct evidence that, depending on the dose, temsirolimus and its analogs may act either as proemetic (at low doses) or antiemetic (at high doses), with the high dose of 20 mg/kg temsirolimus significantly blocking vomiting caused by diverse emetogens. The main limitation of this study was that the molecular mechanisms by which high doses of temsirolimus result in low emesis remain to be deciphered.

The effects of varying doses of temsirolimus and its analogs are discussed in the following sections:

### 4.2 Emetic Effect of Temsirolimus and Its Analogs

Incremental doses of temsirolimus caused emesis in a dose-dependent and bell-shaped manner, with a maximal efficacy of 3.14 ± 0.74 vomits in 78.6% of shrews at its 10 mg/kg dose. Its 20 mg/kg dose evoked less emesis in 25% of the tested shrews, whereas its 40 mg/kg caused no vomiting albeit at a sedative dose which visibly attenuated shrew motor behavior. Clinically, temsirolimus is available as a concentration of 25 mg/ml (Seruga et al., 2009; Fujisaka et al., 2010) administered intravenously (i.v.), which corresponds to 0.42 mg/kg body weight of an adult weighing 60 kg. The calculated HED (human equivalent dose) (Nair and Jacob 2016) corresponding to the shrews’ dose of 20 mg/kg temsirolimus was 0.90 mg/kg, which was 2.14 times higher than the human cancer therapeutic dose for a body weight of 60 mg/kg (0.42 mg/kg). As the intravenous route avoids the process of absorption, temsirolimus (25 mg, i.v.) achieves high peak blood levels in patients rapidly (Boni et al., 2009). In fact, the metabolite of temsirolimus, sirolimus (Atkins et al., 2004), was observed in the blood within 15 min of temsirolimus administration and reached a peak concentration by the end of infusion (Boni et al., 2009). In contrary, following i.p. injection, drugs generally reach peak blood levels relatively more slowly. This can be explained by the fact that following i.p. injection, the primary route of absorption is the mesenteric vessels, which drain into the portal vein and pass through the liver before reaching systemic circulation (Lukas et al., 1971). We speculated that temsirolimus and its analogs administered intraperitoneally in the least shrews could be subjected to first-pass metabolism as well. This first-pass metabolism through the liver may significantly reduce the bioavailability of the drug as showed with oral rapamycin, which is poorly soluble and undergoes extensive first-pass metabolism (Boni et al., 2009). This implied that an optimal antiemetic dose of temsirolimus could be reached in the least shrews with lower doses if it was administered via the i. v. route. Moreover, since least shrews are among the smallest mammals, their physiological systems operate at extremes (Churchfield, 1990) with high mass-specific metabolic rates and turnover times which would, relatively, more rapidly metabolize temsirolimus and its analogs (McNab, 1991).

### 4.3 Effect of Temsirolimus on Locomotor and Rearing Behaviors

Since during the emesis dose-response observations we had noticed that the 40 mg/kg dose of temsirolimus may reduce shrew motor activity, we tested the effect of its lower doses on shrew open-field behaviors. Although, both tested doses of temsirolimus (10 or 20 mg/kg, i p.) did not significantly affect spontaneous locomotor activity (i.e., distance moved), they attenuated the mean rearing frequency in a U-shaped manner with a significant reduction at 10 mg/kg (65.20% versus vehicle; *p* < 0.05). From a pharmacological point of view, the dissociation of ambulation from rearing is not unusual (Lever et al., 2006). Moreover, increased anxiety in rodents can increase rearing behavior without affecting the locomotor activity (Camargo et al., 2021), whereas anxiolytic drugs such as buspirone may reduce rearing behavior at doses that do not affect the ambulatory activity (Panickar and McNaughton 1991). Since temsirolimus can attenuate anxiogenic-like effects of Δ^9^-THC (Puighermanal et al., 2013), one explanation for the attenuated rearing behavior in shrews injected with 10 mg/kg temsirolimus could be due to a high level of anxiety prior and/ or during emesis. However, this will need to be investigated further.

Our overall data provided evidence for the safe use of temsirolimus as an antiemetic drug at 20 mg/kg in shrews.

In addition, with the 20 mg/kg dose, the latency value of temsirolimus was 4.5 times greater than that of rapamycin, suggesting a quicker metabolism of rapamycin. Rapamycin and its analogs possess a central macrolide chemical structure (described in Figure 1), but they differ in their functional group C40. Everolimus and ridaforolimus are hydroxyethyl ester and dimethyl phosphate derivatives, respectively, of rapamycin that are biochemically active without modification. In contrast, temsirolimus is a prodrug of sirolimus (rapamycin) that requires the removal of the dihydroxy methyl propionic acid ester group C40 after the injection to become sirolimus in its active form (MacKeigan and Krueger, 2015). We speculated that this conversion may be a contributing factor to the prolonged latency at 20 mg/kg temsirolimus.

### 4.4 Antiemetic Effects of Temsirolimus Against Diverse Emetogens

We investigated the antiemetic potential of a 20 mg/kg dose of temsirolimus against various doses of diverse emetogens, each being administered at their own maximal effective dose known to evoke emesis in all tested shrews. Temsirolimus reduced to varying degrees both the frequency and percentage of shrews vomiting caused by

1) the nonselective D_2_ receptor agonist apomorphine (2 mg/kg, i.p.) and the more selective and potent D_2_ receptor agonist, quinpirole (2 mg/kg, i.p.) (Darmani et al., 1999; Darmani and Crim 2005). The dopamine D_2_ receptor-preferring antagonist sulpiride has been shown by our laboratory to completely prevent apomorphine-induced vomiting in least shrews at 2 mg/kg (s.c.) (Darmani et al., 1999), but sulpiride (up to 8 mg/kg, s.c.) could not completely protect the shrews from emesis evoked by quinpirole (2 mg/kg, i.p.) (Darmani et al., 1999). Similarly, the antiemetic GSK-3β inhibitor AR-A014418 was found to be less efficacious against quinpirole-evoked emesis, compared with apomorphine (Zhong and Darmani 2020). However, AR-A014418 still significantly decreased the percentage of vomiting (by 55.6 and 83.3% at its 10 and 20 mg/kg doses, respectively) in response to an emetic dose of quinpirole (Zhong and Darmani 2020). In the present study, temsirolimus (20 mg/kg) was slightly more potent against quinpirole-induced vomiting than against that induced by apomorphine. Indeed, pretreatment with temsirolimus (20 mg/kg, i.p.) significantly attenuated the mean vomiting frequency (by 68.85 and 81.80%, respectively) and percentage of shrews vomiting (by 71.43 and 75.0%, respectively) evoked by a 2 mg/kg dose of either apomorphine or quinpirole. Mechanistically, we have previously demonstrated the involvement of the PI3K/mTOR/Akt signaling pathway in dopamine D_2_ receptor-mediated vomiting (Belkacemi et al., 2021). Thus, it is possible that alteration of the PI3K/mTOR/Akt signaling is one of the pathways that is inhibited by the high dose of temsirolimus, but this remains to be demonstrated.

2) the peripherally acting nonselective serotonin 5-HT_3_ receptor agonist, 5-HT (Darmani et al., 2014) or the more selective peripherally/centrally acting 5-HT_3_ receptor agonist, 2-methyl-5-HT, each administered at 5 mg/kg (i.p). Pretreatment with the first-generation selective serotonin 5-HT_3_ receptor antagonist tropisetron (2.5 mg/kg) can attenuate the frequency of vomiting caused by 2-methyl-5-HT in the least shrew by 79% (Darmani et al., 2011). The second generation, and more selective and potent 5-HT_3_ receptor antagonist palonosetron, also decreased the mean vomiting frequency of 2-methyl-5-HT-evoked emesis by 60% at the low dose of 0.1 mg/kg, but only its 10 mg/kg dose could completely block the induced vomiting in least shrews (Darmani et al., 2014). In the present study, temsirolimus (20 mg/kg, i.p.) significantly reduced the vomiting frequency (by 94.73%) and the percentage of shrews vomiting (by 83.33%) in response to the 5-HT (5 mg/kg, i.p.) challenge. Then again, the same dose of temsirolimus reduced the vomiting frequency only by 41.2% and failed to affect the percentage of shrews vomiting in response to the 2-methyl-5-HT challenge. Thus, although relative to selective 5-HT_3_ receptor-antagonists, temsirolimus appeared less efficacious in preventing vomiting in least shrews evoked by 2-methyl-5-HT, our results were consistent with some of the other published data demonstrating that the frequency of 2-methyl-5-HT-stimulated vomiting was sensitive to the inhibitory effect of a 10 mg/kg dose of the broad-spectrum antiemetic, the neurokinin NK_1_ receptor antagonist CP99,994, which, like temsirolimus, failed to alter the percentage of least shrews vomiting (Darmani et al., 2011).

3) the nonselective cholinergic agonist pilocarpine- and the muscarinic-preferring M_1_ agonist McN-A-343 used at 2 mg/kg (i.p). The pharmacology of McN-A-343 was initially described in 1961 by Roszowski (Roszkowski 1961). At first, the agonist was identified as a selective muscarinic M_1_ receptor in the sympathetic ganglia, but later, it was found that McN-A-343 acts as a partial agonist with similar affinity for all five muscarinic receptor subtypes and its relative selectivity depended on a higher efficacy at the M_1_ (and M_4_) subtypes. Because of its relatively high efficacy at the M_1_ receptors, it is now prevalently utilized to differentiate responses mediated through M_1_ receptors from those operating through M_2_ or M_3_ muscarinic receptor subtypes, especially in the CNS (Darmani et al., 2014). Recently, our laboratory has shown that large doses (10 and 20 mg/kg) of the GSK-3β inhibitor, AR-A014418, significantly protects against pilocarpine- and McN-A-343-induced emesis, whereas only a lower dose of the more potent GSK-3αβ inhibitor SB216763 (0.25 mg/kg, i.p.), was required for the suppression of vomiting evoked by these cholinergic agonists (Zhong and Darmani 2020). In line with AR-A014418 findings, our present results indicate that temsirolimus (20 mg/kg) significantly decreased the vomiting frequency and percentage (60 and 70%, respectively) of shrews vomiting evoked by pilocarpine and McN-A-343. Collectively, these data support the antiemetic efficacy of temsirolimus against pilocarpine- and McN-A-34-evoked emesis.

4) the selective substance P neurokinin NK_1_ receptor agonist GR73632 (5 mg/kg) which causes robust vomiting through the activation of substance P neurokinin NK_1_ receptors in least shrews (Darmani et al., 2008; Ray et al., 2009c). The selective and more potent NK_1_ receptor antagonist netupitant at 10 mg/kg can completely protect shrews from GR73632 (5 mg/kg)-evoked vomiting (Zhong et al., 2019). Other inhibitors such as LTCC blockers amlodipine (50% protection at 10 mg/kg) and nifedipine (complete protection at 5 mg/kg), and GSK-3 inhibitors AR-A014418 (80% protection at 10 mg/kg) and SB216763 (70% protection at 0.25 mg/kg) also suppress vomiting caused by GR73632 in a dose-dependent manner (Zhong et al., 2014; Zhong and Darmani 2020). In the present study, we found that the 20 mg/kg dose of temsirolimus reduced both the frequency (70%) and the percentage of least shrews vomiting (56%) following the GR73632 challenge.

5) the selective LTCC agonist FPL64176 used at 10 m/kg. LTCC regulates extracellular Ca^2+^ influx into the cytosol (Zamponi et al., 2015). FPL64176 is an extracellular Ca^2+^- mobilizing agent that caused vomiting in all tested shrews at a 10 mg/kg dose (Darmani et al., 2014; Zhong et al., 2014). In the present study, the 20 mg/kg dose of temsirolimus completely protected the shrews from vomiting induced by FPL64176 (10 m/kg). Our results imply that temsirolimus may act by preventing the influx of extracellular Ca^2 +^, thereby protecting the shrews from vomiting. The fact that temsirolimus treatment can improve hypercalcemia induced by parathyroid hormone-related peptide (PTHrP) (Okui et al., 2010), lends support for a role for temsirolimus as a Ca^2+^ blocker. Recently, our laboratory has found that FPL64176 causes emesis through the Ca^2+^-induced Ca^2+^ release (CICR) process by an initial influx of extracellular Ca^2+^ through LTCCs followed by the release of intracellular-stored Ca^2+^ from the endoplasmic reticulum via ryanodine receptors (RyRs) (Zhong et al., 2018). The induced intracellular Ca^2+^ mobilization was followed by the activation of some intracellular emetic signaling kinases such as ERK1/2, PKCα/βII, and Akt. Hence, we speculate that temsirolimus may also alter the FPL6417–evoked emesis by deactivating ERK1/2, PKCα/βII, or Akt kinases. However, this hypothesis will require further investigation.

6) the specific inhibitor of the sarco/endoplasmic reticulum Ca^2+^-ATPase (SERCA) pump thapsigargin used at 0.5 mg/kg. The SERCA pump transports free cytosolic Ca^2+^ into Ca^2+^ stores in the lumen of the endoplasmic reticulum (ER) to counterbalance the cytosolic intracellular Ca^2+^ released from the ER into the cytoplasm through the inositol trisphosphate (IP_3_) receptors and ryanodine (RyR) receptors localized on the ER membrane (Garaschuk et al., 1997; Gomez-Viquez et al., 2003). Thapsigargin may also cause a rapid elevation in cytosolic Ca^2+^ concentrations through discharge of intracellular Ca^2+^ stores from the ER into the cytosol (Jackson et al., 1988; Thastrup et al., 1990). Activation of the extracellular Ca^2+^ influx implicates various Ca^2+^ channels present in the cell membrane including store-operated Ca^2+^ entry (SOCE) and LTCCs (Parekh and Putney 2005; Feske 2007). Thapsigargin evokes vomiting (Zhong et al., 2016) by triggering an initial elevation in the cytoplasmic Ca^2+^ concentration by inhibiting the SERCA as well as releasing Ca^2+^ from the ER into the cytoplasm via both RyR- and IP_3_-receptors (IP_3_Rs). This is followed by an extracellular Ca^2+^ influx through LTCCs prior to the intracellular activation of emetic signals (CaMKII and ERK1/2) and an increase in the c-fos immunoreactivity in the brainstem emetic nuclei (AP, NTS, and DMNX). In the present study, we showed that temsirolimus reduces the frequency of evoked vomiting by 94.14% and protects 80% of the shrews from thapsigargin-induced vomiting. Thus, it is possible that temsirolimus may prevent emesis by countering the intracellular effects of thapsigargin on Ca^2+^ stores in the ER. However, this hypothesis warrants further testing.

7) the cannabinoid CB_1_ receptor-selective inverse agonist/antagonist SR141716A (20 mg/kg, i.p.). SR141716A can induce emesis in the least shrew at large doses (20–40 mg/kg, i.p.) which is sensitive to cannabinoid CB_1_ receptor agonists including Δ^9^-THC (Darmani 2001). In the present study, temsirolimus at 20 mg/kg completely protected the shrews from SR 141716A (20 mg/kg, i.p.)-evoked emesis. Large emetic doses of SR141716A increased both the brainstem tissue level and turnover of emetic neurotransmitters, dopamine and serotonin (Darmani et al., 2003). Based on the present and published findings (Belkacemi and Darmani 2020), we speculate that perhaps the 20 mg/kg dose temsirolimus may block the release of serotonin and dopamine and/or their downstream signaling which subsequently prevent emesis by mechanisms yet to be discovered.

8) the cytotoxic chemotherapeutic agent cisplatin (10 mg/kg, i.p.). Cisplatin produces vomiting biphasically in both humans and other vomit-competent species (Rudd et al., 1996; Tanihata et al., 2003; Jordan et al., 2007; Darmani et al., 2009). In humans, chemotherapy-induced nausea and vomiting are classified as acute, happening within the first 24 h, or delayed, taking place after the first 24 h (Jordan et al., 2007; Darmani et al., 2013; Jordan et al., 2016). The clinical management of chemotherapy-induced nausea and vomiting caused by high-dose cisplatin include a combination of antiemetics including a 5-HT_3_ receptor antagonist (e. g., palonosetron), an NK_1_ receptor antagonist (e. g., netupitant) (Darmani et al., 2015; Jordan et al., 2016; Rudd et al., 2016), and a glucocorticoid such as dexamethasone. Similarly, our present findings demonstrate that temsirolimus by itself can attenuate the acute phase of cisplatin-induced vomiting by 57% during the 2 h post-administration period. This is a significant finding since, as described previously, a triple regimen of antiemetics is required to reduce high-dose cisplatin-evoked emesis. Unlike temsirolimus, the GSK-3 inhibitors SB216763 and AR-A014418 have failed to significantly affect cisplatin (10 mg/kg, i.p.)-induced acute emesis (0–4 h following injection) (Zhong and Darmani 2020).

Collectively, our results demonstrated that a high dose of temsirolimus has a broad-spectrum antiemetic efficacy of varying degrees against diverse emetogens. It probably suppresses emesis by blocking the downstream signaling of specific key emetic receptors (D_2_, 5-HT_3_, CB_1_R, M_1_, and NK_1_), Ca^2+^ channel regulators, and possibly, SP and 5-HT release in the case of cisplatin.

### 4.5 Central Site of Emetic Action of Temsirolimus

c-fos protein induction is a classical tool for the evaluation of the neuronal activation of post-agonist stimulation (Bullitt 1990). c-fos immunohistochemistry has proven valuable for finding the sites of action of the emetic drugs such as cisplatin (Reynolds et al., 1991). A c-fos immunoreactivity pattern has also been observed in several vomit-competent species including ferrets after loperamide-induced vomiting (Bhandari et al., 1992; Zaman et al., 2000), and in shrews following the administration of diverse emetogens (Darmani et al., 2008; Zhong et al., 2016; Zhong et al. 2018; Zhong et al. 2019; Belkacemi et al., 2021). Thus, we used immunohistochemistry to determine c-fos responsiveness in the shrew brainstem following the injection of varying doses of temsirolimus (0, 10, and 20 mg/kg, i.p.). A 10 mg/kg emetic dose of temsirolimus resulted in the induction of c-fos-immunoreactive nuclei in the AP (∼2.0-fold) and NTS (3.9-fold), but not in the DMNX. This indicates that 1) peripheral mediators (e.g., serotonin or dopamine) released into the blood may have a significant role in temsirolimus-induced emesis since the AP-zone frequently produces c-fos-immunoreaction related to the activation by blood-borne mediators (Ray et al., 2009a), and 2) temsirolimus and its analogs may cross the brain–blood barrier and reach the AP and NTS emetic nuclei (Zhao et al., 2012). Specifically, temsirolimus has demonstrated to cross the brain–blood barrier in preclinical models (Zhao et al., 2012) and everolimus can penetrate the brain in both rats and mice (O'Reilly et al., 2010). The presence of c-fos in the NTS is not surprising since the medial subnucleus of the NTS (mNTS) is the key integrative site for central nervous system modulation of the emetic reflex. Also, the NTS receives emesis-related information both from the central nervous system (AP and cerebral cortex) (Hornby 2001), and the peripheral emetic loci from the gastrointestinal tract (Wood et al., 1999), conveyed by vagal and splanchnic afferents and integrates these signals that could result in the exacerbated c-fos expression in vomiting animals. The lack of induction of c-fos with the 20 mg/kg temsirolimus dose, supports our behavioral data demonstrating a lack of significant vomiting at this dose.

## 5 Conclusion

Temsirolimus and its analogs are promising cancer therapeutics for various types of human malignancies, but little is known concerning their emetic effects. We found that the intraperitoneal administration of temsirolimus causes vomiting in a dose-dependent but bell-shape manner with maximal efficacy at 10 mg/kg. Its 20 mg/kg and probably larger doses may have broad-spectrum antiemetic effects. Although neither its 10 nor 20 mg/kg doses affected the spontaneous locomotor activity in the least shrews, its 10 mg/kg dose did significantly reduce the mean rearing frequency in a U-shaped manner. Temsirolimus analogs everolimus, ridaforolimus, and rapamycin also produced similar bell-shaped dose-response emetic effects. Consistent with our emesis data, although c-fos expression in the shrew brainstem emetic nuclei was significantly increased in the AP and NTS after the administration of the maximally effective emetic dose (10 mg/kg) of temsirolimus, its larger antiemetic dose (20 mg/kg, i.p.) had no significant effect. The limitation of this study was that it was extremely difficult to obtain sufficient blood to determine accurate levels of these drugs from least shrews which were 3–5 g in weight. Moreover, our laboratory was not equipped to determine the concentration levels of these drugs. Despite these limitations, our study clearly demonstrated that the 20 mg/kg dose of temsirolimus exhibited broad-spectrum antiemetic efficacy against an array of centrally and peripherally acting receptor-selective and -nonselective emetogens. As the 20 mg/kg temsirolimus did not affect the locomotor activity or rearing behaviors, it may be also beneficial to expand the pharmacological armamentarium of temsirolimus in cancer patients and possibly its analogs as an antiemetic.

## Data Availability

The raw data supporting the conclusion of this article will be made available by the authors, without undue reservation.

## Abbreviations

AP, area postrema; NTS, nucleus tractus solitarius; DMNV, dorsal motor nucleus of the vagus; mTOR, mammalian targets of rapamycin; 5-HT3R, serotonergic type-3 receptor; NK1R, neurokinin 1 receptor; i.p., intraperitoneally; 5-HT, serotonin; SERCA, sarco/endoplasmic reticulum Ca2+-ATPase; and LTCC, L-type calcium channel.
