# Supplementary material for: Evidence for Bell-Shaped Dose-Response Emetic Effects of Temsirolimus and Analogs: The Broad-Spectrum Antiemetic Efficacy of a Large Dose of Temsirolimus Against Diverse Emetogens in the Least Shrew (Cryptotis parva)
Source: Front Pharmacol. 2022 Apr 4;13:848673. doi: 10.3389/fphar.2022.848673 (PMC9014009; doi:10.3389/fphar.2022.848673)
Supplement: Supplementary file 1 [file Table1.pdf]

Table S1. Summary of the antiemetic effect of temsirolimus against vomiting induced by various emetogens

Groups of drugs tested	Drug concentration and route	Number of animals within a group (N)	% Vomit frequency and P-value	Shrews vomiting (%) and P-value
Dopamine receptor agonists	**Apomorphine**<br>(a dopamine $D_{2/3}$ non selective agonist; 2 mg/kg, i.p.)	6 – 7	68.85<br>*P = 0.04	71.43<br>**P = 0.008
	**Quinpirole**<br>(a dopamine D_2 selective agonist; 2 mg/kg, i.p.)	8	81.80<br>*P = 0.01	75<br>**P = 0.002
Serotonergic 5-HT_3 receptor agonists	**5-HT**<br>(serotonin, a peripherally-acting non-selective 5-HT_3 receptor agonist; 5mg/kg. i.p.)	6	94.73<br>***P = 0.0001	83.33<br>**P = 0.003
	**2-Methyl-5-HT**<br>(a centrally/peripherally-acting and selective agonist; 5 mg/kg, i.p.)	6	58.33<br>*P = 0.01	100<br>P > 0.05
Cholinergic M_1 receptor agonists	P**ilocarpine**<br>(a nonselective agonist, 2 mg/kg, i.p.)	5	84.48<br>**P < 0.002	60<br>*P = 0.04
	**McN-A-343**<br>(a selective agonist; 2 mg/kg, i.p.)	10	71.80<br>*P = 0.02	70<br>**P = 0.001
Tachykininergic Neurokinin 1 (NK_1) receptor agonist	**GR73632**<br>(a selective agonist, 5 mg/kg, i.p.)	7 – 9	69.96<br>**P = 0.006	55.56<br>*P < 0.05
L-type Ca^{2+} channel (LTCC) agonist	F**PL64176** (10 mg/kg, i.p.)	5 – 6	100<br>***P = 0.001	100<br>**P = 0.003
Sarco/endoplasmic reticulum (ER) Ca^{2+} ATPase inhibitor (SERCA)	**Thapsigargin** (0.5 mg/kg, i.p.)	5 – 6	94.14<br>**P < 0.01	80.00<br>**P = 0.006
Cannabinoid CB_1 receptor antagonist/inverse agonist	**SR141716A** (20 mg/kg, i.p.)	7 – 11	100<br>***P = 0.0001	100<br>****P < 0.0001
Chemotherapeutic agent	**Cisplatin** (10 mg/kg, i.p.)	7 – 8	80.48<br>**P = 0.002	57.14<br>*P = 0.02

Asterisk indicates significance vs. Vehicle
