# Supplementary material for: Evidence for Bell-Shaped Dose-Response Emetic Effects of Temsirolimus and Analogs: The Broad-Spectrum Antiemetic Efficacy of a Large Dose of Temsirolimus Against Diverse Emetogens in the Least Shrew (Cryptotis parva)
Source: Front Pharmacol. 2022 Apr 4;13:848673. doi: 10.3389/fphar.2022.848673 (PMC9014009; doi:10.3389/fphar.2022.848673)
Supplement: Supplementary file 2 [file Table2.pdf]

Table S2: Comparison of mean vomit time latency between temsirolimus and rapamycin among the vomiting animals in each tested group

**Dose/ Mean latency value of drug (sec) ± SEM**	**Temsirolimus** (Mean ± SEM)	**Rapamycin** (Mean ± SEM)	**Fold change = (mean latency of temsirolimus (sec)/ mean latency of rapamycin (sec)**
5 mg/kg, i.p.	242 ± 41.0	412 ± 113.8	0.6
10 mg/kg, i.p.	181 ± 40.4	164 ± 57.3	1.1
20 mg/kg, i.p.	540 ± 210.7	120 ± 0	4.5
